# 

**Published:** 1893-09-23

**Authors:** 


PRICE TWOPENCE.

No. 365, Vol. XVI.—Sept. 23rd, 1893.

[Registered at the General Post Office as a Newspaper for transmission in the United Kingdom and Abroad.]

SEPTEMBER 23RD

WITH EXTRA NURSING SUPPLEMENT

Per Annum, 8s. 6d., post free.

Monthly Parts, 6d.; post free, 8d.

Ditto per Annum, 8s., post free.

### THE HOSPITAL

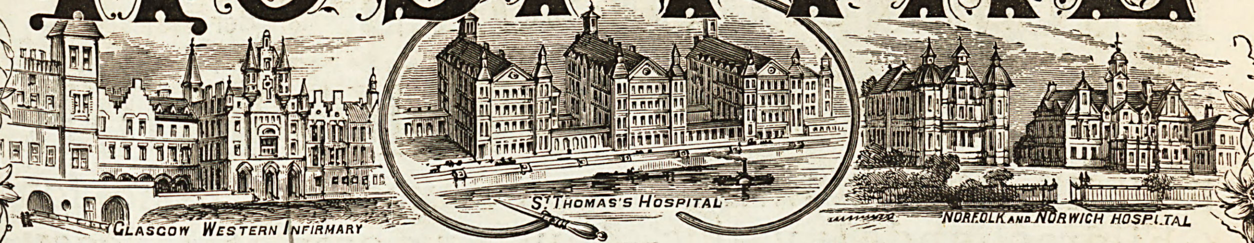

WITH EXTRA NURSING SUPPLEMENT.

No. 365. Vol. XIV. SATURDAY, SEPTEMBER 23RD, 1893. [TWOPENCE.

